# Early-Life Exposure to Non-Absorbable Broad-Spectrum Antibiotics Affects the Dopamine Mesocorticolimbic Pathway of Adult Rats in a Sex-Dependent Manner

**DOI:** 10.3389/fphar.2022.837652

**Published:** 2022-06-30

**Authors:** Camila González-Arancibia, Victoria Collio, Francisco Silva-Olivares, Paula Montaña-Collao, Jonathan Martínez-Pinto, Marcela Julio-Pieper, Ramón Sotomayor-Zárate, Javier A. Bravo

**Affiliations:** ^1^ Laboratorio de Neuroquímica y Neurofarmacología, Centro de Neurobiología y Fisiopatología Integrativa (CENFI), Instituto de Fisiología, Facultad de Ciencias, Universidad de Valparaíso, Valparaíso, Chile; ^2^ Grupo de NeuroGastroBioquímica, Instituto de Química, Facultad de Ciencias, Pontificia Universidad Católica de Valparaíso, Valparaíso, Chile; ^3^ Programa de Doctorado en Ciencias Mención Neurociencias, Facultad de Ciencias, Universidad de Valparaíso, Valparaíso, Chile; ^4^ Programa de Magíster en Ciencias Médicas, Mención Biología Celular y Molecular, Universidad de Valparaíso, Valparaíso, Chile

**Keywords:** antibiotics, gut microbiota, dopamine, dopamine receptors, VTA, NAcc, CPP

## Abstract

Gut microbiota with a stable, rich, and diverse composition is associated with adequate postnatal brain development. Colonization of the infant’s gut begins at birth when parturition exposes the newborn to a set of maternal bacteria, increasing richness and diversity until one to two first years of age when a microbiota composition is stable until old age. Conversely, alterations in gut microbiota by diet, stress, infection, and antibiotic exposure have been associated with several pathologies, including metabolic and neuropsychiatric diseases such as obesity, anxiety, depression, and drug addiction, among others. However, the consequences of early-life exposure to antibiotics (ELEA) on the dopamine (DA) mesocorticolimbic circuit are poorly studied. In this context, we administered oral non-absorbable broad-spectrum antibiotics to pregnant Sprague-Dawley dams during the perinatal period (from embryonic day 18 until postnatal day 7) and investigated their adult offspring (postnatal day 60) to assess methylphenidate-induced conditioned place preference (CPP) and locomotor activity, DA release, DA and 3,4-dihydroxyphenylacetic acid (DOPAC) content in ventral tegmental area (VTA), and expression of key proteins within the mesocorticolimbic system. Our results show that ELEA affect the rats conduct by increasing drug-seeking behavior and locomotor activity induced by methylphenidate of males and females, respectively, while reducing dopamine striatal release and VTA content of DOPAC in females. In addition, antibiotics increased protein levels of DA type 1 receptor in prefrontal cortex and VTA of female rats, and tyrosine hydroxylase in VTA of adult male and female rats. Altogether, these results suggest that ELEA alters the development of the microbiota-gut-brain axis affecting the reward system and the response to abuse drugs in adulthood.

## 1 Introduction

In mammals, gut colonization begins at birth, and several factors such as type of birth (cesarean or vaginal), diet (breastfeeding or infant formula), and antibiotics exposure (maternal or neonatal), among others, affects the infant gut microbiota composition, in a window of time related to postnatal nervous system development (Cryan and Dinan, 2012;O'Mahony et al., 2014; Diaz Heijtz, 2016; Delara et al., 2021). For example, studies in germ-free (GF) animals (animals completely devoid of microbes) have shown an increase in locomotor activity (Davis et al., 2016; Schretter et al., 2018), a reduction in social interactions associated with spending more time in self-grooming that suggests an increase in repetitive behaviors (Desbonnet et al., 2014). At cellular levels, GF mice have hypermyelinated axons in the prefrontal cortex (PFCx) compared to control animals (Hoban et al., 2016b). However, GF animals have no clinical correlate to what happens in nature, nor clinical settings. An approach to this, however, is the use of antibiotics. These compounds are commonly used in the clinical practice to treat infections in newborns, or their mothers, even in the immediate postnatal stage (Bourgeois et al., 2016; Cotten, 2016; Lavebratt et al., 2019; Ran et al., 2021). Thus, antibiotics provide an effective pharmacological tool to disrupt gut colonization during early-life. Arentsen et al. (2017) showed that administration of ampicillin (0.6 mg/ml in the drinking water) to pregnant mice from 5 days before parturition until 3 days post-partum prevented the translocation of bacterial peptidoglycan (PGN) from the intestinal lumen into the brain of the offspring (Arentsen et al., 2017). The bacterial PGN in the central nervous system (CNS) has been associated with a reduction of c-Met expression, which is related to a higher risk of autism (Arentsen et al., 2017). In this context, maternal antibiotic exposure increased cerebral c-Met expression in the offspring (Arentsen et al., 2017), affecting normal infant brain development. Undoubtedly, early-life exposure to antibiotics (ELEA) affects normal gut colonization (altering diversity and richness), having the potential of impacting brain development, increasing the vulnerability to neuropsychiatric-related disorders such as addiction or drug dependence.

Drug dependence is a neuropsychiatric disorder characterized by a compulsion to seek and take the drug, loss of control in limiting intake, and negative emotional state during withdrawal (American Psychiatric Association, 2013). At the neurobiological level, the main neural system involved in the effect of addictive drugs is the reward system (mesocorticolimbic circuit) (Koob and Volkow, 2010). This system is a group of neural structures that mainly comprise dopaminergic projections from the ventral tegmental area (VTA) to nucleus accumbens (NAcc) and PFCx (Volkow and Fowler, 2000; Koob and Volkow, 2010), which are physiologically activated in response to natural rewards (i.e., food, sex, and social interaction) and supra-physiologically activated by abuse drugs (Bassareo and Di Chiara, 1997; Frohmader et al., 2010). These high NAcc DA extracellular levels activate postsynaptic DA type 1 receptor (D_1_) that potentiates synaptic transmission and early gene expression (i.e., c-Fos, Arc, NAC-1, between others) (Nestler, 2001). Furthermore, chronic substance abuse promotes overexpression of NAcc *Δ*FosB, increasing NF-κB, GluR2, and Cdk5 expressions, playing a crucial role in developing and maintaining addiction (Nestler et al., 2001). On the other hand, several factors of vulnerability to addiction have been studied, such as genetic predisposition (i.e., low mRNA expression of DA type 2 receptor [D_2_]), the type of drug abused, and psychological factors (child abuse, chronic exposure, and stress), among others (Kreek et al., 2005; Shumay et al., 2012; Everitt and Robbins, 2016; Belles et al., 2021). However, the role of altered gut microbiota on drug dependence has not been fully studied. In this context, Kiraly et al. showed that administration of a cocktail of broad-spectrum non-absorbable antibiotics (0.5 mg/ml bacitracin, 2 mg/ml neomycin, 0.2 mg/ml vancomycin and 1.2 μg/ml pimaricin) in drinking water for 14 days, increased conditioned place preference (CPP) to cocaine (5 mg/kg) in adult C57Bl/6j mice (Kiraly et al., 2016). Also, exposition to antibiotics produced alterations in the NAcc expression pattern of some receptors such as TrkB, D_1_, and GluA (Kiraly et al., 2016). On the other hand, chronic drug use could affect the gut microbiota and contribute to the maintenance of addictive behavior. For example, chronic exposure to cocaine decreases microbial richness and diversity in the gut (Scorza et al., 2018), while methamphetamine exposure also causes a decrease in bacterial metabolites that interact directly with the CNS (i.e., short-chain fatty acids) in methamphetamine-induced CPP rats (Ning et al., 2017).

New evidence has recently shown that alterations in gut microbiota composition affects brain functionality, especially in midbrain dopaminergic circuits. In this context, administration of a non-absorbable broad-spectrum antibiotics cocktail, before and after a lesion with 6-hydroxydopamine in the adulthood reduces dopaminergic neuron loss and improved motor deficit (Koutzoumis et al., 2020). However, the long-term neurochemical and behavioral consequences associated with mesocorticolimbic circuits have not been fully studied in an animal model of ELEA. To address this and considering that the main source of gut microbes to newborns is the maternal intestinal tract, we lowered the maternal gut microbial diversity and richness through an oral administration of a broad-spectrum cocktail of antibiotics. This mixture of antibiotics was given to the dam from embryonic day (ED) 18 until postnatal day (PD) 7, and the effects of such intervention were studied in the female and male offspring at PD60. In these animals, we evaluated the effects of ELEA on drug conditioning susceptibility and locomotor activity induced by methylphenidate (MPH), a DA transporter (DAT) blocker. We also assessed *in-vivo* dorsolateral striatal DA release, as well as quantified DA and 3,4-dihydroxyphenylacetic acid (DOPAC) tissue content in VTA. Finally, we analyzed expression of key proteins (D_1_, D_2_, DAT, and tyrosine hydroxylase [TH]) in PFCx, NAcc, and VTA.

## 2 Materials and Methods

### 2.1 Reagents

DA and 3,4-dihydroxyphenylacetic acid (DOPAC) standards, EDTA, and 1-octanesulfonic acid were purchased from Sigma-Aldrich, Inc. (St. Louis, Missouri, United States). Methylphenidate hydrochloride was donated by Laboratorios Andrómaco S.A. (ISPCH N° F-20582/18, Peñalolén, Santiago, Chile). All other reagents were of analytical and molecular grade.

### 2.2 Animals

A total of 124 animals were used in the present study. 90-day-old female Sprague-Dawley pregnant dams (*n* = 12) were treated with the following broad-spectrum non-absorbable antibiotics cocktail administered through oral gavage from ED18 until PD7: 100 mg/kg neomycin, 100 mg/kg bacitracin, 100 mg/kg vancomycin and 5 μg/kg pimaricin (ELEA, *n* = 6) or vehicle (1 ml/kg of saline solution 0.9% NaCl; control, *n* = 6). Pups were weaned at PD21 and housed in a temperature- and humidity-controlled room (22 ± 2°C; 50 ± 5%, respectively) under artificial illumination (12-h light/12-h dark; light on at 08:00 a.m.), with food (Prolab^®^ RMH 3000, LabDiet, St. Louis, MO, United States) and water *ad libitum*. All experiments were performed in adult male (control male, n = 31; ELEA male, n = 22) and female (control female, n = 27; ELEA female, n = 32) offspring, at a median age of PD60; MPH-induced CPP was carried out between PD57 and PD63, DA protein analysis in NAcc at PD60, and mesolimbic DA and DOPAC content were performed at PD63. All experimental procedures were approved by the Bioethics and Biosafety Committees of the Universidad de Valparaíso, Pontificia Universidad Católica de Valparaíso and National Agency of Research and Development (ANID-Chile) through FONDECYT program. All efforts were made to reduce the number of animals used and to minimize animal suffering. For neurochemical and cellular experiments, animals were rapidly decapitated with a guillotine for small animals (model 51330, Stoelting™ Co., Wood Dale, IL, United States).

### 2.3 Conditioned Place Preference

Rats used for CPP were assigned to the following experimental groups: control male-saline (*n* = 6), control male-MPH (*n* = 10), control female-saline (*n* = 5), control female-MPH (*n* = 6), ELEA male-saline (*n* = 5), ELEA male-MPH (*n* = 5), ELEA female-saline (*n* = 9), and ELEA female-MPH (n = 10). The features of the CPP apparatus and the protocol used were previously described (Bonansco et al., 2018; Velasquez et al., 2019). Briefly, the conditioning protocol consisted of three parts: pre-test (1 day before the conditioning period), conditioning, and test (24 h after the last injection). For the pre-test and the test, rats were placed in the neutral-gray center compartment with both guillotine doors open and were allowed to explore the entire apparatus (mid gray area and two outer compartments, black and white) for 15 min. The time spent in each compartment was measured by analyzing the recordings obtained by internet protocol cameras (LX-C202 model; Lynx Security, China) fixed above each place preference apparatus and wireless connected to a computer in another room. During the conditioning period (5 days), the non-preferred compartment (the white side) was associated with reward induced by MPH injection at a dose of 5 mg/kg i. p., once per day, which was previously stablished elsewhere (Kashefi et al., 2021). The preferred compartment (black side), as well as the animals that did not receive drug during the conditioning period, were paired with a 1 ml/kg saline i. p. injection. Afterward, conditioning/preference was determined as the percentage of change between the time spent in the white compartment on the test day and the pre-test day.

### 2.4 Locomotor Activity

Basal, saline- and MPH-induced locomotor activities were measured in a different cohort of ELEA and control rats. During the first 30 min after each animal was placed in the arena (50 cm long, 50 cm height, and 50 cm wide), video recording was taken, and basal locomotor activity was established. At 30 min, saline solution (1 ml/kg i. p.) was injected and locomotor activity was recorded for another 60 min. At 90 min, a single dose of MPH (5 mg/kg i. p.) was injected and locomotor activity was recorded for an additional 60 min. Recording was carryout with wireless cameras (model LX-C202, Lynx Security, China) fixed 1.5 m above each arena and connected to a computer in another room *via* wifi. Videos were analyzed using ANY-Maze™ video tracking system (Stoelting™ Co., Wood Dale, IL, United States), measuring the total distance traveled (m) every 5 min. Arenas were wiped and cleaned with 5%v/v ethanol solution after each trial.

### 2.5 Neurochemical Studies

#### 2.5.1 Fast Scan Cyclic Voltammetry

24 h after locomotor activity/open field protocol, rats were deeply anesthetized with isoflurane (3% in 0.8 L/min air flow) in an induction chamber for 3 min and placed in a stereotaxic apparatus (model 68002, RWD Life Science Co. Ltd., Shenzhen, China) with a mask to maintain anesthesia for all the experiment (isoflurane 1.5% in 0.8 L/min air flow), using an animal anesthesia system (model 510, RWD Life Science Co. Ltd., Shenzhen, China). Body temperature was maintained at 37°C with a water circulation system (model 68662, RWD Life Science Co. Ltd., Shenzhen, China). Rats were exposed to a craniotomy for the implantation of three electrodes. A glassy-carbon microelectrode (working electrode) was implanted in dorsal striatum using the coordinates from the Rat Brain Atlas (Paxinos and Watson, 2009) (dorsal striatum: 1.3 mm posterior, 2.5 mm lateral, and 4.0 mm ventral to brain surface) and an Ag/AgCl reference electrode was positioned in the contralateral cortex. The electrode potential was linearly scanned (−0.4–1.2 V and back to −0.4 V vs. Ag/AgCl) and cyclic voltammograms were assessed at the carbon fiber electrode every 100 ms with a scan rate of 400 V/s using a voltammeter/amperometer (model Chem-Clamp Potensiostat, Dagan Corporation, Minneapolis, MN, United States). A bipolar stimulating electrode (model MS 303/2A, Plastics one Inc., Roanoke, VA, United States) was implanted in the midbrain using the coordinates from the Rat Brain Atlas (Paxinos and Watson, 2009) (Regarding to bregma: 5.2 mm posterior, 1.3 mm lateral, and 7.5 mm ventral to brain surface). Phasic DA release was stimulated with the following parameters: monophasic +, 60 pulses, 60 Hz, 4 ms, 300 μA (current stimulus isolator NL800A; Digitimer, Ltd., Hertfordshire, United Kingdom). For data collection, two National Instruments acquisition cards (NI-DAQ; PCI-6711 and PCI-6052e; National Instruments, Austin, TX, United States) were used to interface the potentiostat and stimulator with Demon Voltammetry and Analysis software (Wake Forest Health Sciences, Winston-Salem, NC, United States) [42]. Data collection began once electrical stimulation in the midbrain induced a suitable dopamine peak in the striatum. Phasic DA was stimulated every 5 min and three steady baseline collections were measured. After a basal period, a saline injection (1 ml/kg, i. p.) and DA release were measured for another 15 min. Finally, rats received an injection of MPH (5 mg/kg i. p.) and phasic DA release was assessed for 75 min after drug administration. After the experiment, animals were killed by rapid decapitation with a guillotine for small animals (model 51330, Stoelting™ Co., Wood Dale, IL, United States). Brain was immediately extracted and placed on an ice-cold surface. Then, NAcc, VTA and striatum were micro-dissected at 4°C using a micro-punch (Harris Micro-Punch™, 2.0 mm of diameter, Ted Pella Inc., CA, United States). The tissues were then weighed and stored at −80°C for further analysis. Data were analyzed with Demon Voltammetry and Analysis software using peak height, area, and tau as parameters of DA release induced by electrical stimulation and uptake kinetics.

#### 2.5.2 Dopamine and DOPAC Content in NAcc and VTA

Once the CPP protocol was finalized, saline-injected control (males, *n* = 5; females, *n* = 5) and ELEA (males, *n* = 5; females, *n* = 9) rats were used for analyzing DA and DOPAC content in NAcc and VTA. Animals were euthanized 2 h after CPP test phase was finished (at PD63). Brain was immediately extracted and placed on an ice-cold surface. NAcc and VTA were micro-dissected at 4 °C using a brain matrix (model 68711, RWD Life Science, Shenzhen, P.R. China) and micro-punch (model 15076; diameter 2.0 mm, Harris Uni-Core, Ted-Pella Inc., Redding, CA, United States) as described previously (Bonansco et al., 2018; Guajardo et al., 2020). Briefly, NAcc and VTA were weighed on an analytical balance (model JK-180, Chyo balance corp, Tokyo, Japan) and homogenized in 400 μl of 0.2 N perchloric acid using a sonicator (model XL2005, Microson Ultrasonic Cell Disruptor, Heat Systems, Farmingdale, NY, United States). The homogenate was centrifuged to 12,000×*g* for 15 min at 4°C (model Z233MK-2, Hermle Labor Technik GmbH, Wehingen, Germany) and the supernatant was filtered (model EW-32816-26; 0.2 μm, HPLC Syringe Filters PTFE, Cole-Parmer, Instrument Company, Vernon Hills, IL, United States). The final supernatant was injected to HPLC coupled with an electrochemical detector. The pellet of each sample was resuspended in 1 ml of 1M NaOH for protein quantification by the Bio-Rad Protein Assay (Bio-Rad Laboratories, Inc., Richmond, CA, United States), using bovine serum albumin as standard, and the readout was performed in a microplate spectrophotometer (Epoch™, BioTek Instruments Inc., Winooski, VT, United States). Ten microliters of supernatant were injected to the HPLC-ED system with the following equipment: an isocratic pump, (model PU-2080 Plus, Jasco Co. Ltd., Tokyo, Japan), a C18 column (model Kromasil 100-3.5-C18, AkzoNobel, Bohus, Sweden), and an electrochemical detector (set at 650 mV, 0.5 nA; model LC-4C, BAS, West Lafayette, IN, United States). The mobile phase, containing 0.1 M NaH_2_PO_4_, 1.0 mM 1-octanesulfonic acid, 1.0 mM EDTA, and 8.0% (^v^/_v_) CH_3_CN (pH 3.4) was pumped at a flow rate of 0.125 ml/min. DA and DOPAC levels were assessed by comparing the respective peak area and elution time of the sample with a reference standard and the quantification was performed using a calibration curve for each neurotransmitter (Program ChromPass, Jasco Co. Ltd., Tokyo, Japan). The concentration of DA and DOPAC was expressed as pg per mg of protein.

### 2.6 Western Blot

For protein determinations, a new cohort of rats that were not exposed to any other experiments was used: Control (males, *n* = 4; females, *n* = 4) and ELEA (males, *n* = 4; females, *n* = 5). Animals were euthanized and the brain was immediately extracted and placed on an ice-cold surface to extract NAcc, VTA and PFCx, using a brain matrix and micro-punch. The tissues were then weighed and stored at −80°C for further analysis. To determine D_1_, D_2_, DAT, Phosphorylated DAT (pDAT) and TH protein levels in NAcc, VTA and PFCx, brain areas were homogenized using RIPA buffer (pH = 8.0, 150 mM, NaCl, 50 mM Tris-HCl, 1%^v^/_v_ Nonidet P40, 0.1% ^w^/_v_ SDS, 2 mM EDTA, 1.5 mM PMSF, and a protease inhibitor cocktail [Cat# G6521, Promega™]) using a sonicator (model XL 2005, Microson Ultrasonic CellDisruptor, Heat Systems, United States). The total protein concentration was determined using the Bio-Rad Protein Assay (Bio-Rad Laboratories, Inc., Richmond, CA, United States), using bovine serum albumin as standard, and the readout was performed in a microplate spectrophotometer (Epoch™, BioTek Instruments Inc., Winooski, VT, United States). Thirty micrograms of total protein from each sample were separated by 10% SDS-PAGE. Proteins were transferred to nitrocellulose membranes (Cat# 88018, 0.45 μm pore, Thermo Scientific™, Rockford, IL, United States) at 350 mA for 1.5 h. Non-specific sites of membrane binding were blocked with 5% skim milk (for D_2_, pDAT, DAT, and TH) or with 5% bovine serum albumin (BSA, for D_1_) in TTBS (0.1% Tween-20, 20 mM TBS, 137 mM NaCl) for 1 h at room temperature. Later, nitrocellulose membranes were incubated overnight at 4°C with primary antibodies diluted in blocking solution. The antibodies used in this study were: rabbit anti-D_1_ diluted 1:1,000 (Cat# AB1765P, MerckMillipore, Merck KGaA, Darmstadt, Germany), rabbit anti-D_2_ diluted 1:1,000 (Cat# AB5084P, MerckMillipore, Merck KGaA, Darmstadt, Germany), anti-DAT diluted 1:2,000 (Cat# 434-DATEL2, PhosphoSolutions, Denver, CO, United States), rabbit anti-phosphorylated DAT (pDAT) diluted 1:1,000 (Cat# p435-53, PhosphoSolutions, Denver, CO, United States), TH diluted 1:5,000 (Cat# 657012, Calbiochem, MerckMillipore, Merck KGaA, Darmstadt, Germany) and rabbit anti-GAPDH diluted 1:10,000 (Cat# ab9485, Abcam, Cambridge, MA, United States) as constitutive protein. Membranes were washed three times with T-TBS and then incubated during 1 h at room temperature in blocking solution with the secondary antibody for D_1_ and D_2_, diluted 1:5,000; for DAT and pDAT, diluted 1:10,000; for TH, diluted 1:20,000; and for GAPDH, diluted 1:20,000 Peroxidase-conjugated AffiniPure F (ab’)_2_ Fragment Donkey anti-rabbit (Cat# 711-036-152, Jackson Inmuno Research, laboratories, Inc., West Grove, PA, United States). For chemiluminescent detection, we used SuperSignal™ West Dura Extended Duration Substrate (Cat# 34075, Thermo Fisher Scientific, Waltham, MA, United States) and the images of the membranes were obtained using a benchtop transilluminator (EpiChemi3 Darkroom, UVP, Upland, CA, United States). The images were analyzed using ImageJ™ software (http://rsbweb.nih.gov/ij/).

### 2.7 Statistical Analysis

Data were expressed as mean ± SEM. Two-way ANOVA followed by post-hoc Tukey test (multiple comparisons) was performed for the data analysis from CPP, DA content and protein expression, considering sex and ELEA or drug administration and ELEA as variables. Three-way ANOVA, followed by post-hoc Tukey test (multiple comparisons) was performed for FSCV data analysis, locomotor activity and cumulative locomotor activity, considering three variables: time, sex, and ELEA. The statistical analyses were carried out with GraphPad Prism version 9.2.0 (GraphPad Software, San Diego, CA, United States), and *p* < 0.05 was considered statistically significant.

## 3 Results

### 3.1 Weight Gain is not Affected by Perinatal Exposure to Non-Absorbable Broad-Spectrum Antibiotics

**FIGURE 1:**
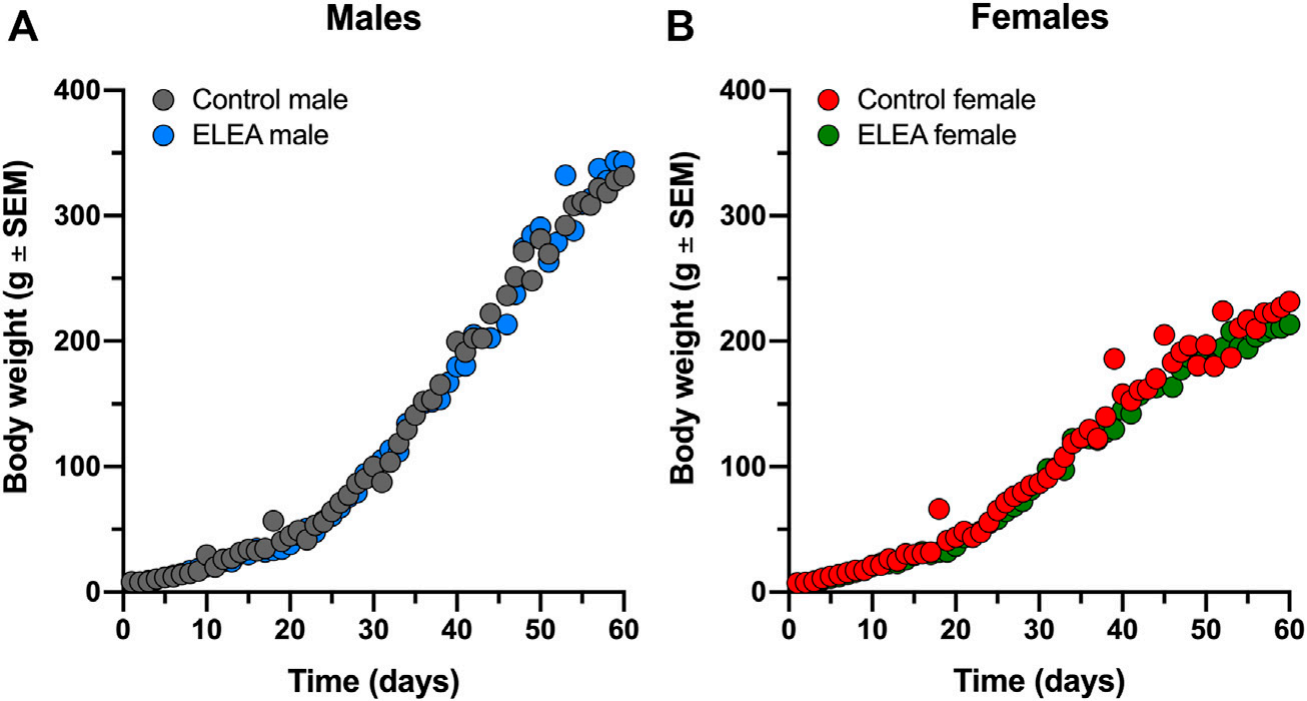
ELEA did not affect body weight of male and female rats. There are no differences in weight gain within **(A)** male and **(B)** female rats perinatally exposed to antibiotics (grey) and their respective controls (black). Animals were weighed from birth until PD63 (control males, *n* = 31; ELEA males, *n* = 22; control females, *n* = 27; ELEA females, *n* = 32).

Pregnant Sprague-Dawley dams were treated with a non-absorbable broad-spectrum antibiotics cocktail (neomycin, bacitracin, vancomycin and pimaricin) from ED18 until PD7, via oral gavage. Offspring weight gain was monitored from birth to adulthood (PD1 to PD63). We did not find significant differences between experimental groups (Figure 1). Overall, male rats gain more weight than female rats, as expected.

### 3.2 ELEA Alters Drug-Seeking Behavior in Rats in a Sex-Dependent Manner

**FIGURE 2:**
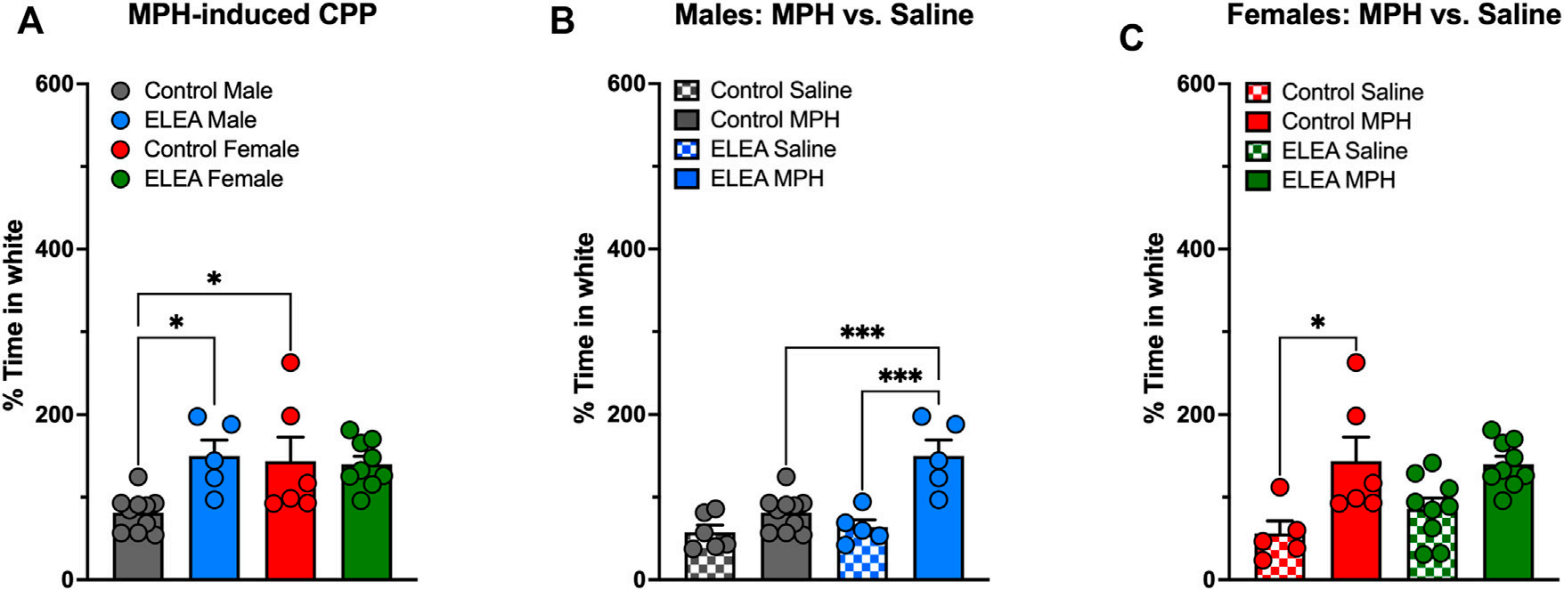
Methylphenidate preference of adult male and female rats exposed to antibiotics during early life. ELEA increased time spent in white compartment (associated with drug delivery) only in males and not females **(A–C)** after a 5-days conditioning period to MPH. Also, control females spent more time in the MPH-associated compartment than control males **(A)**. ELEA males spent more time in the white compartment when paired with drug delivery compared to saline injection and MPH injection in control animals **(B)**. Conversely, only control females spent more time in the white compartment when paired with MPH delivery compared to saline injection, an effect not observed in ELEA females **(C)** (control male-saline, *n* = 6; control male-MPH, *n* = 10; ELEA male-saline, *n* = 5; ELEA male-MPH, *n* = 5; control female-saline, *n* = 5; control female-MPH, *n* = 6; ELEA female-saline, *n* = 9; ELEA female-MPH, *n* = 10; * = *p* < 0.05; *** = *p* < 0.001).

We evaluated drug-seeking behavior in adult male and female rats exposed to antibiotics early in life and compared them to their respective controls (Figure 2). 2-way ANOVA analysis showed a significant interaction between antibiotic exposure and sex (F_(1,26)_ = 5.502, *p* = 0.0269) and a main effect of antibiotic exposure (F_(1,26)_ = 4.426, *p* = 0.0452) when MPH-induced CPP males and females were analyzed (Figure 2A). Also, Tukey’s multiple comparisons post-test reveals that ELEA males spend more time in the white compartment (associated with MPH administration) than control males (*p* = 0.0231, Figure 2A), and that control females spend more time in the MPH-associated compartment than control males (*p* = 0.0299, Figure 2A). When analyzing MPH and saline conditioning in males and females separately, 2-way ANOVA showed significant main effect of ELEA (F_(1,22)_ = 8.319, *p* = 0.0086) and drug exposure (F_(1,22)_ = 25.75, *p* < 00,001), as well as a significant interaction between the two variables (F_(1,22)_ = 8.319, *p* = 0.0086) of males (Figure 2B). Male rats also showed that ELEA increases time spent in the white compartment when paired with drug administration compared to saline injection (Tukey’s post-test, *p* = 0.0002; Figure 2B) and ELEA males spent significantly more time in the MPH-associated compartment than control males (Tukey’s post-test, *p* = 0.0005; Figure 2B). On the other hand, we only observed a significant drug administration effect (F_(1,25)_ = 17.67, *p* = 0.0003) on females, specifically control females spent more time in the white compartment when paired with MPH administration compared to saline (Tukey’s post-test, *p* = 0.0146; Figure 2C). This result shows that early-life antibiotic exposure affects drug response differently in male and female rats.

### 3.3 ELEA Reduces MPH-Induced Locomotor Activity in Males

**FIGURE 3:**
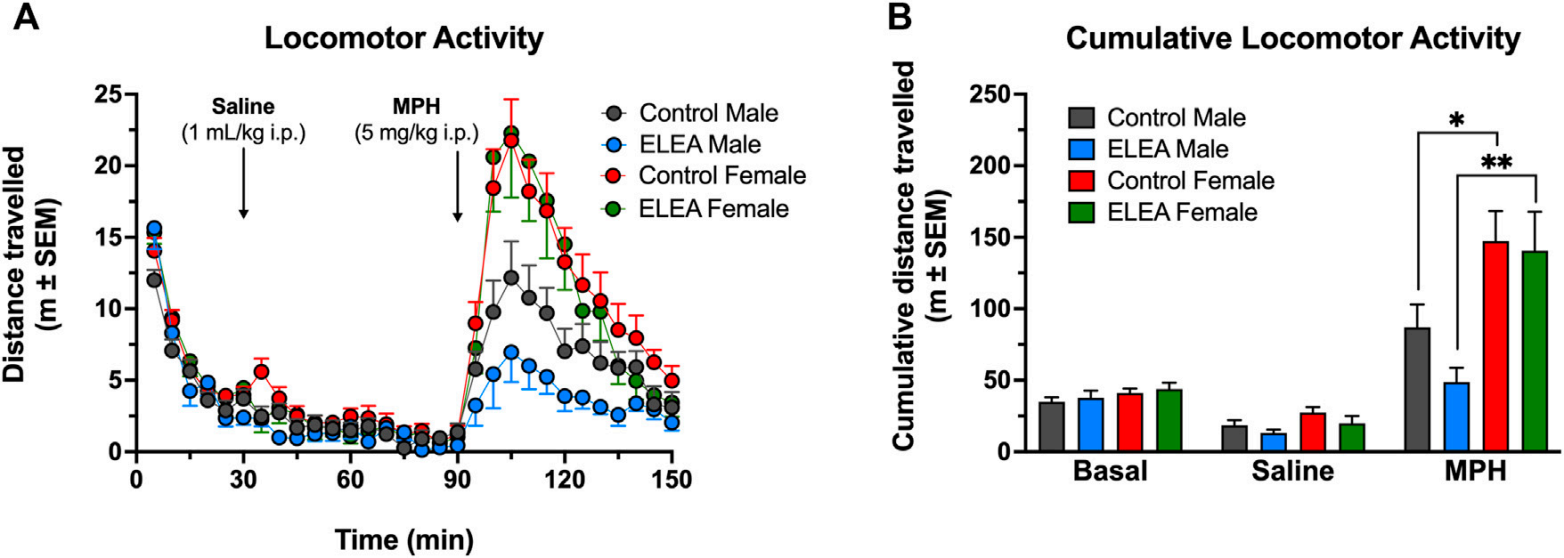
ELEA effects on locomotor activity of adult rats. **(A)** There were no differences in basal locomotor activity nor when rats were injected with saline solution (1 ml/kg, i. p.). 15 min after drug administration (MPH; 5 mg/kg, i. p.) ELEA female rats showed higher locomotor activity than ELEA males (*** = *p* < 0.001). **(B)** Cumulative locomotor activity showed female rats, both control and ELEA animals, travelled significantly more distance than their male counterparts (* = *p* < 0.05; ** = *p* < 0.01). There were no differences in cumulative basal distance travelled, nor when saline was injected (control males, *n* = 11; ELEA males, *n* = 8; control females, *n* = 12; ELEA females, *n* = 8).

To evaluate the effect of ELEA on locomotor activity, a different cohort of male and female rats, were injected with saline (1 ml/kg, i. p.) and an hour later, with MPH (5 mg/kg, i. p.). Figure 3 shows locomotor activity (Figure 3A) and cumulative locomotor activity (Figure 3B). 3-way ANOVA analysis showed significant main effect of antibiotic exposure (Figure 3A: F_(1, 1050)_ = 11.22, *p* = 00,008), time (Figure 3A: F_(29, 1050)_ = 41.47, *p* < 00,001), and sex (Figure 3A: F_(1, 1050)_ = 133.7, *p* < 00,001), with a significant interaction between time and sex (Figure 3A: F_(29, 1050)_ = 7,114, *p* < 00,001). There were no differences in basal locomotor activity nor when rats were injected with saline solution between any of the groups. After 10 min of drug administration, female rats showed higher locomotor activity than males, with ELEA animals showing a greater difference and longer effect in the time range from 100 to 120 min (Figure 3A; Tukey’s multiple comparisons test:100, 105, 110 min, *p* < 00,001; 115 min, *p* = 00,001; and 120 min, *p* = 00,071) than control animals (Figure 3A; Tukey’s multiple comparisons test: 100 min, *p* = 00,113; and 105 min, *p* = 0.001). On the other hand, cumulative locomotor activity (Figure 3B) showed main effects of MPH administration (Figure 3B: F_(2, 70)_ = 60.30, *p* < 00,001) and sex (Figure 3B: F_(1, 35)_ = 16.45, *p* = 00,003), and a significant interaction between these two variables ((Figure 3B: F_(2, 70)_ = 11.18, *p* < 00,001). Tukey’s multiple comparisons post-test revealed the following: 1) control males traveled significantly more distance after drug administration (Figure 3B: Control males, saline vs MPH, *p* = 00,015), an effect than was not observed in ELEA males (Figure 3B: ELEA males, saline vs MPH, *p* = 0,7006); 2) Female rats, both control and ELEA, traveled significantly more distance after drug administration (Figure 3B: Control females, saline vs MPH, *p* < 00,001; ELEA females, saline vs MPH, *p* < 00,001); and 3) under MPH’s effect, female rats traveled a greater distance than their male counterparts (Figure 3B: MPH, control males vs control females, *p* = 00,083; MPH, ELEA males vs ELEA females, *p* = 00,002). There were no differences in cumulative basal distance travelled, nor when saline was injected (Ctrl Females, *n* = 12; ELEA Females, *n* = 8; Ctrl Males, *n* = 11; ELEA Males, *n* = 8).

### 3.4 ELEA Decreases *In-vivo* Striatal Dopamine Release in Females

**FIGURE 4:**
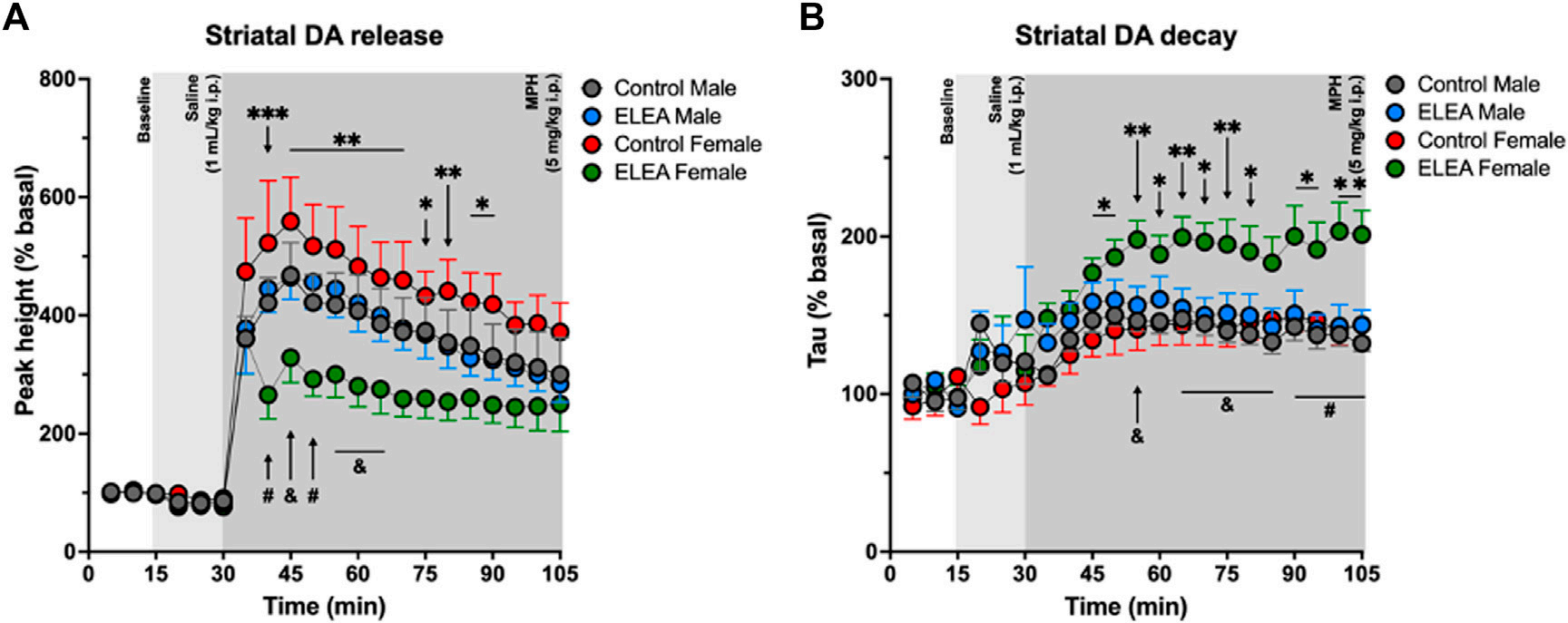
Analysis of the dorsolateral striatal *in-vivo* dopamine release evoked by substantia nigra electrical stimulation. **(A)** Dopamine release regarding the baseline (%) for the peak height. ELEA females release lower amount of dopamine than control females and ELEA males, but antibiotic exposure did not affect DA release in males. **(B)** Striatal dopamine decay. ELEA females showed a higher decay (Tau) than control females and ELEA males. (ELEA female Vs. control female: *** = *p* < 0.001; ** = *p* < 0.01; * = *p* < 0.05. ELEA female Vs. ELEA male: # = *p* < 0.01; and = *p* < 0.05; control male, *n* = 6; ELEA male, *n* = 5; control female, *n* = 4; ELEA female, *n* = 4).

To further explore the effects of ELEA on the dopaminergic system, we evaluated *in-vivo* dorsolateral striatal DA release trough Fast-Scan Cyclic Voltammetry (FSCV) procedures in the same cohort of locomotor activity assessment (24h later). There were no differences in the peak height during basal nor after saline injection (1 ml/kg, i. p.). However, 10 min after MPH administration (5 mg/kg, i. p.), ELEA females showed a significantly lower DA release than control females (Figure 4A, Tukey’s multiple comparison post-test, ELEA females vs control females: 40 min *p* = 0.0004; 45 min *p* = 0.0014; 50 min *p* = 0.0018; 55 min *p* = 0.0034; 60 min *p* = 0.0051; 65 min *p* = 0.0089; 70 min *p* = 0.0054; 75 min *p* = 0.0162; 80 min *p* = 0.0094; 85 min *p* = 0.0241; 90 min *p* = 0.0178), and that ELEA males (Figure 4A, ELEA females vs ELEA males: 40 min *p* = 0.046; 45 min *p* = 0.0316; 50 min *p* = 0.0093; 55 min *p* = 0.0222; 60 min *p* = 0.0266; 65 min *p* = 0.0490). Also, 3-way ANOVA showed significant main effects of ELEA (Figure 4A: F _(1.294)_ = 42.25, *p* < 0.0001) and time (Figure 4A: F _(20, 294)_ = 34.68, *p* < 0.0001), with a significant interaction between sex and ELEA (Figure 4A: F _(1, 294)_ = 45.17, *p* < 0.0001). Representative color plots of basal, saline and MPH (45 min) dopamine release can be seen in supplementary figures (Supplementary Figure S1).

Along with these results, we also observed an increase in Tau value, or striatal DA decay, of ELEA females when compared to control females (Figure 4B, Tukey’s multiple comparison post-test, ELEA females vs control females: 45 min *p* = 0.0329; 50 min *p* = 0.0207; 55 min *p* = 0.0046; 60 min *p* = 0.0289; 65 min *p* = 0.0055; 70 min *p* = 0.0111; 75 min *p* = 0.0081; 80 min *p* = 0.0251; 90 min *p* = 0.0100; 95 min *p* = 0.0250; 100 min *p* = 0.0016; 105 min *p* = 0.0043), and to ELEA males (Figure 4B, Tukey’s multiple comparison post-test, ELEA females vs ELEA males: 55 min *p* = 0.0276; 65 min *p* = 0.0179; 70 min *p* = 0.0142; 75 min *p* = 0.0200; 80 min *p* = 0.0313; 85 min *p* = 0.0309; 90 min *p* = 0.0094; 95 min *p* = 0.0074; 100 min *p* = 0.0016; 105 min *p* = 0.0025), with significant main effects of time (Figure 4B, 3-way ANOVA: F _(20, 315)_ = 11.82; *p* < 0.0001), sex (Figure 4B, 3-way ANOVA: F _(1, 315)_ = 14.82; *p* = 0.0001), and antibiotic exposure (Figure 4B, 3-way ANOVA: F _(1, 315)_ = 60.35, *p* < 0.0001), and significant interactions between time and sex (Figure 4B, 3-way ANOVA: F _(20, 315)_ = 1.928, *p* = 0.0105), and sex and ELEA (Figure 4B, 3-way ANOVA: F _(1, 315)_ = 25.10, *p* < 0.0001). Taken together, these results show that antibiotic exposure during early-life alters the DA circuitry of females but not males, and reveals, once again, sex differences between males and females from ELEA experimental group.

### 3.5 ELEA Decreases VTA DOPAC Content in Adult Female Rats

Considering the differences in TH protein levels in NAcc and VTA of adult rats, due to sex or to perinatal exposure to antibiotics respectively, we evaluated DA and DOPAC content in NAcc and VTA of control and ELEA rats that were injected with saline during CPP conditioning (naïve rats to MPH), 24 h after CPP test. Per experimental conditions (control or ELEA rats), we did not observe statistical differences in DA, DOPAC content, nor DOPAC/DA ratio in NAcc of the adult rats (Supplementary Figure S2).

**FIGURE 5:**
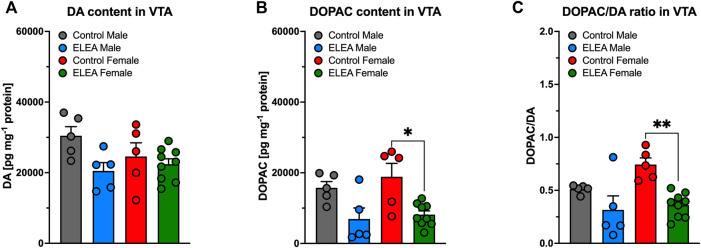
DA and DOPAC content of VTA of early-life antibiotic exposed rats. ELEA did not alter VTA’s DA content of the adult rat **(A)**. However, antibiotic exposed females had lower levels of DOPAC in VTA when injected with saline during CPP conditioning period **(B)**, as well as lower DOPAC/DA ratio **(C)**. (Control male, n = 6; ELEA male, *n* = 5; control female, *n* = 5; ELEA female, *n* = 9; * = *p* < 0.05; ** = *p* < 0.01).

Regarding neurochemical changes in VTA, 2-way ANOVA showed a significant main effect of early-life antibiotic exposure on DA (Figure 5A) and DOPAC (Figure 5B) content, as well as their DOPAC/DA ratio (Figure 5C), of VTA from adult rats (F_(1, 20)_ = 5.772, *p* = 0.0261; F_(1, 20)_ = 17.05, *p* = 0.0005; and F_(1,20)_ = 17.45, *p* = 0.0005; respectively). However, Tukey’s post-test only revealed a reduction on VTA’s DOPAC content and DOPAC/DA ratio in ELEA female rats (Figures 5B,C: *p* = 0.0132; *p* = 0.0024, respectively).

### 3.6 ELEA Alters D_1_ and D_2_ Receptors in the Adult Mesocorticolimbic System

**FIGURE 6:**
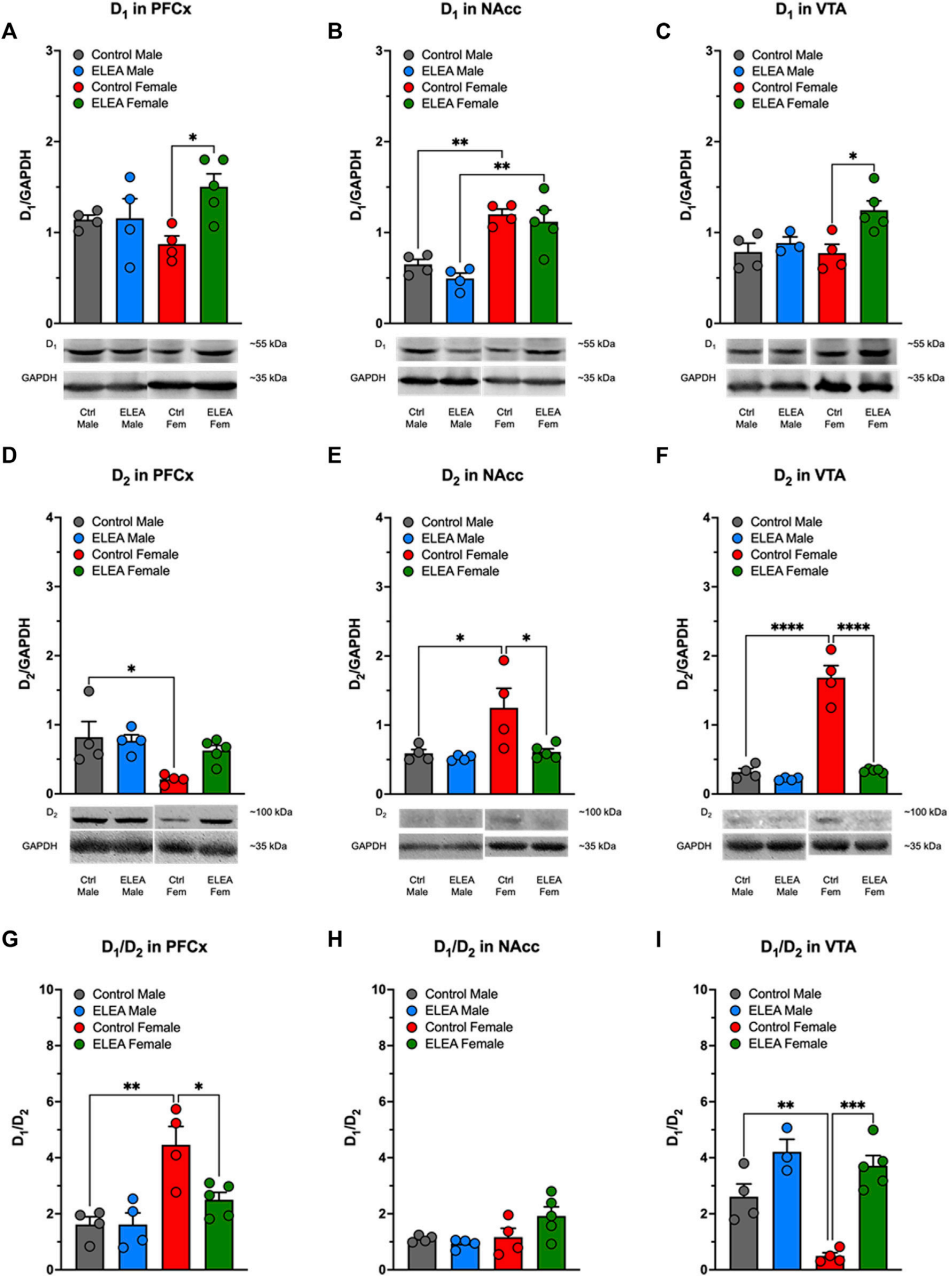
Effect of perinatal antibiotic exposure on the protein levels of the mesocorticolimbic dopaminergic system. Early life antibiotic exposure increased D1 protein levels of PFCx **(A)** and VTA **(C)** in adult female rats. Both control and antibiotic exposed females had higher levels of D1 in NAcc than their male counterparts **(B)**. PFCx D2 protein levels were higher in control males than in control females **(D)**. In both, NAcc **(E)** and VTA **(F)**, control females had higher D2 protein level than control males, but antibiotic exposure during early life decreased protein expression. PFCx D1/D2 ratio was higher in control females than control males, but antibiotic exposure decreased the ratio in females **(G)**. There were no significant changes in D1/D2 ratio of NAcc **(H)**. VTA D1/D2 ratio was lower in control female than in control male, but antibiotic exposure increased the ratio of female rats **(I)**. (Control male, *n* = 4; ELEA male, *n* = 4; control female, *n* = 4; ELEA female, *n* = 5; * = *p* < 0.05; ** = *p* < 0.01; *** = *p* < 0.001; **** = *p* < 0.0001).

Animals used to determine D_1_, D_2_, DAT, p-DAT, and TH protein levels in PFCx, NAcc and VTA, were not previously exposed to MPH or any other DAT blocker (drug-naïve animals). We found significant differences in D_1_ protein levels in PFCx and VTA due to antibiotic exposure (Figures 6A,C, 2-way ANOVA: F_(1,13)_ = 5.281, *p* = 0.0388; and F_(1,12)_ = 8.216, *p* = 0.0142, respectively). Tukey’s post-test indicated that D_1_ protein levels increased in both nuclei of females, but not males (Figures 6A,C: *p* = 0.0278 and *p* = 0.0169, respectively). Also, D_1_ protein levels showed a significant difference between sexes (Figure 6B: F_(1,13)_ = 42.16, *p* < 0.0001) in NAcc of control and antibiotic exposed rats (Figure 6B, control male vs control female: *p* = 0.0051; and ELEA male vs ELEA female: *p* = 0.0012). Regarding D_2_ receptor protein levels, we observed a significant sex-related difference in PFCx D_2_ (Figure 6D: F_(1,13)_ = 9.176, *p* = 0.0097), where control female rats had a significantly lower protein level than male control animals (*p* = 0.0217). On the other hand, we observed a significant sex-associated difference (Figure 6E: F_(1,13)_ = 7.343, *p* = 0.0179) and treatment effect (Figure 6E: F_(1,13)_ = 6.504, *p* = 0.0242) on NAcc D_2_ protein levels. Female control rats had significantly higher receptor expression than control males (Figure 6E: *p* = 0.0259) and ELEA females (Figure 6E: *p* = 0.0231) rats. Interestingly, we observed that VTA D_2_ protein levels have significant differences due to sex and antibiotic exposure (Figure 6F: F_(1,13)_ = 74.95, *p* < 0.0001; F_(1,13)_ = 71.09, *p* < 0.0001; respectively). Also we observed a significant interaction between the two variables (Figure 6F: F_(1,13)_ = 52.63, *p* < 0.0001). In addition, we found that female control rats have higher VTA D_2_ protein levels than male control rats (Figure 6F, *p* < 0.0001). However, perinatal antibiotic exposure significantly decreased the VTA D_2_ protein levels (Figure 6F, *p* < 0.0001).

When we evaluated the D_1_/D_2_ ratio in PFCx, NAcc and VTA, we found significant differences with regards to sex and antibiotic exposure in PFCx (Figure 6G: F_(1,13)_ = 19.88, *p* = 0.0006; and F_(1,13)_ = 5.520, *p* = 0.0353; respectively), as well as a significant interaction between the two variables (Figure 6G: F_(1,13)_ = 5.510, *p* = 0.0354), where control female rats have higher D_1_/D_2_ ratio than control male rats (Figure 6G: *p* = 0.0021). However, ELEA decreased this ratio significantly in females when compared to control animals (Figure 6G: *p* = 0.0211). In addittion, we did not observed significant changes in D1/D2 ratio in NAcc (Figure 6H). On the other hand, we observed significant sex-related difference and antibiotic exposure effect on the VTA D_1_/D_2_ ratio (Figure 6I: F_(1,12)_ = 12.11, *p* = 0.0045; and F_(1,12)_ = 41.23, *p* < 0.0001; respectively), in which control female rats showed a significant lower ratio than control male rats (Figure 6I: *p* = 0.0076). In contrast, perinatal antibiotic exposure significantly increased this ratio (Figure 6I: *p* = 0.0001).

### 3.7 ELEA did not Affect DAT and pDAT Protein Levels in the Adult Mesocorticolimbic System

The pDAT, total DAT (phosphorylated and non-phosphorylated) and the pDAT/DAT ratio in PFCx, NAcc and VTA were not affected by perinatal antibiotic exposure (Supplementary Figure S3).

### 3.8 ELEA Increases TH Protein Levels in VTA of Adult Female Rats

**FIGURE 7:**
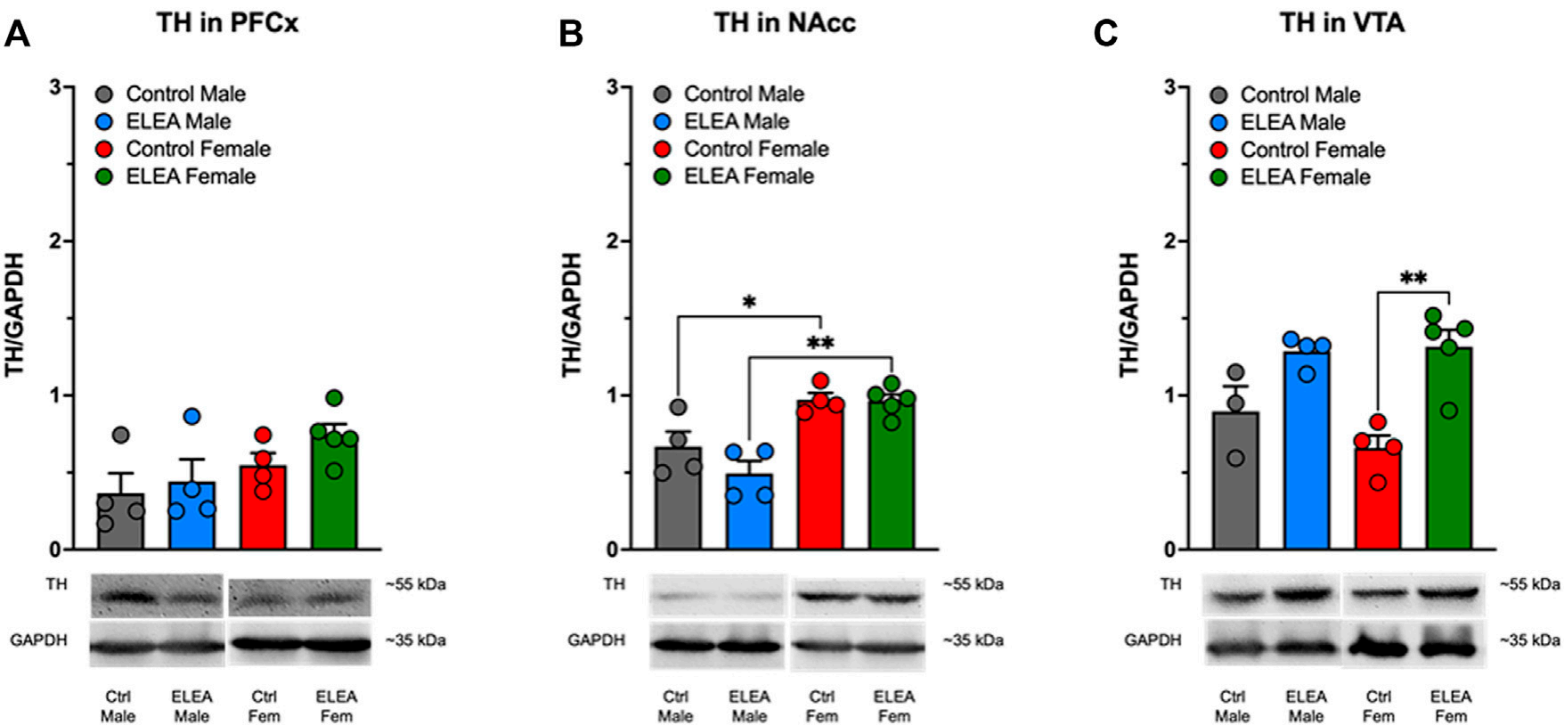
Effect of perinatal antibiotic exposure on TH protein levels of the mesocorticolimbic dopaminergic system. Early life antibiotic exposure had no effect on TH expression levels in PFCx **(A)**. Both control and antibiotic exposed females had higher TH protein levels than their male counterparts in NAcc **(B)**. Perinatal antibiotic exposure increased TH expression in VTA of male and female rats **(C)**. (Control male, *n* = 4; ELEA males, *n* = 4; control female, *n* = 4; ELEA female, *n* = 5; * = *p* < 0.05; ** = *p* < 0.01).

We did not find significant differences in PFCx TH protein levels of male and female animals (Figure 7A). However, we observed a sex-associated difference in NAcc TH protein levels (Figure 7B: F_(1,13)_ = 32.68, *p* < 0.0001), where both control and ELEA female rats have higher TH protein levels than their respective male counterparts (Figure 7B, control male vs control female rats: *p* = 0.0378; ELEA male vs ELEA female rats: *p* = 0.0011). Finally, antibiotic exposure had a main effect on TH expression levels in VTA (Figure 7C: F_(1, 12)_ = 25.10, *p* = 0.0003), where ELEA increased VTA TH protein levels in adult female rats (Figure 7C: *p* = 0.0022).

## 4 Discussion

Early-life exposure to broad-range non-absorbable antibiotics has been previously shown to decrease diversity and richness of gut microbiota in adult rodents (Clarke et al., 2014; O'Mahony et al., 2014), suggesting that perinatal exposure to antibiotics alters gut microbiota composition in adulthood. Additionally, these findings showed that reducing the diversity and richness of gut microbiota during key stages of development, results in different metabolic and neuronal alterations (Clarke et al., 2014; O'Mahony et al., 2014; Mueller et al., 2015; Codagnone et al., 2018; Noye Tuplin et al., 2021), which could also be affecting the neurodevelopment and function of the NAcc and, possibly, of the whole dopaminergic reward system impacting drug response.

In the present study, we show that ELEA alters the drug seeking behavior of the adult offspring, moreover, in a sex-dependent manner. Specifically, we observed an enhanced response in the methylphenidate (MPH) induced CPP in ELEA males, but not in females (Figure 2). The data presented here suggest that ELEA promotes sex-dependent effects on the mesocorticolimbic system of the rat, which may be associated to changes in gut microbiota composition. Several authors have demonstrated that the antibiotic cocktail used here alters gut microbiota composition, exerting several effects on the dopaminergic system (Kiraly et al., 2016; Koutzoumis et al., 2020). Kiraly et al. (2016) administered a similar antibiotic cocktail, in different doses (Neomycin 2 mg/ml; Bacitracin 0.5 mg/ml; Vancomycin 0.2 mg/ml; and Pimaricin 1.2 μg/ml), to adult male C57BL6/j mice in their drinking water for 7–10 days and observed an enhanced sensitivity to cocaine in the CPP protocol. In addition, Kiraly et al. (2016) found increased BDNF, GluA2 and D_1_ mRNA expression in NAcc, showing that alterations in the gut microbiota composition alter the transcriptional regulation of genes related to the reward system and drug response. Also, those results were exclusively due to a local non-absorbable antibiotic effect within the gut, as mice injected intraperitoneally with the antibiotic cocktail did not show any of these changes (Kiraly et al., 2016). Present results partly agree with those findings, strongly suggesting that gut symbionts impact on dopaminergic circuitry in rodents in adulthood. Moreover, the data here presented suggest that transmission of an altered gut microbiota from mother to newborn impacts on the offspring’s adult behavior and neurochemistry, particularly regarding the mesocorticolimbic circuit.

On the other hand, Koutzoumis et al. (2020), also used a similar antibiotic cocktail, in the same doses and administration method as Kiraly et al. (2016), though they employed a 6-hydroxydopamine (6-OHDA) lateral lesion Parkinson’s disease (PD) rat model. They gave the antibiotic cocktail to adult male Sprague Dawley rats 2 weeks prior to the 6-OHDA lesion and up to 12 weeks post-lesion, to evaluate gut microbiota composition’s effect on PD dopaminergic system. They found that the antibiotic treatment attenuates DA neuron degeneration in substantia-nigra of 6-OHDA animals, improved motor deficits and decreases pro-inflammatory markers in striatum tissue from the 6-OHDA injected-antibiotic treated animals (Koutzoumis et al., 2020). Taken jointly, these findings indicate that gut microbiota does in fact alter, and further regulates, dopaminergic function in the brain. The issue related to possible pathways and connections between the gastrointestinal tract and the reward system was thoroughly reviewed (Gonzalez-Arancibia et al., 2019). However, the cited studies, as well as our own, are only associative and not causative. Therefore, further, and deeper gut microbiota analysis are required.

When we analyzed DA and DOPAC content in NAcc and VTA areas obtained from male and female adult rats that went through CPP behavioral test, we found that DOPAC content in VTA decreases in ELEA females, while there are no changes in DA content (Figure 5). This could indicate that DA metabolism is altered, which could account for the differences observed in both behavior and proteins, since a decrease in DOPAC could indicate greater availability of DA. However, this is not the case because DA content remained unchanged (Figure 5). It is worth mentioning that the machinery necessary to metabolize DA is not exclusively located in pre- and post-synaptic neurons, but also in the periphery of the synapse (Hansson, 1984), so when obtaining the samples by micro-punch, we cannot differentiate where specifically the DOPAC reduction is occurring. Finally, the reduction of DOPAC may be occurring due to an increase in COMT activity, which could be elucidated by assessing HVA levels.

Moreover, ELEA alters the expression of key proteins within the dopaminergic reward system in the adult rat, also in a sex-dependent manner. We observe that drug-naïve ELEA adult females exhibit increased D_1_ protein levels in PFCx and VTA (Figures 6A,C, respectively), and higher TH protein expression in VTA (Figure 7C), together with a reduction of D_2_ expression in NAcc and VTA (Figures 6E,F, respectively), while ELEA did not alter protein expression in males (Figures 6A–F). Mesocorticolimbic protein content changes in ELEA females may suggest increased sensitivity to DAT blockers, however, MPH-induced CPP revealed that ELEA females do not respond to the 5 mg/kg dose of MPH as control females (Figure 2). These differences could be occurring by modulation of drug response by other neuronal populations, such as the glutamatergic system, which abundantly innervates the mesocorticolimbic circuit (Gonzalez-Arancibia et al., 2019), and alterations of the host’s gut microbiota by antibiotics, in addition to drug exposure, increases mRNA expression of GluA2 in NAcc of mice (Kiraly et al., 2016), suggesting that glutamate response to drugs may be altered in antibiotic exposed animals, or that MPH is affecting glutamatergic circuitry impacting the drug-seeking behavior, as MPH also regulates glutamate receptors activity and transcript levels within the PFCx (Cheng et al., 2014; Salman et al., 2021). Further studies are needed to test this hypothesis.

Sex differences in gut microbiota-manipulated animals during DA system critical development periods have been reported recently by Noye Tuplin et al. (2021), where they supplemented 21-days old rats with maternal milk oligosaccharides, which are known to regulate infant’s gut microbiota during lactation period and are considered as prebiotics. They found that oligosaccharide-supplemented females displayed lower levels of DAT mRNA in VTA, and lower D_2_ mRNA expression in NAcc (Noye Tuplin et al., 2021), suggesting that gut microbiota manipulation during the mesolimbic development period alters the resulting adult dopaminergic system differentially according to sex. Taken together, these results suggest that gut microbiota composition regulates protein expression within the dopaminergic reward system during critical developmental periods, and that sex is an important factor to determine how these regulations occur.

Part of the results that we have obtained in our paper may be related to a long-term increase in glucocorticoids induced by ELEA, because treatment of adult mice with antibiotics for 3–4 weeks increases plasma corticosterone (Wu et al., 2021). Furthermore, in adult male rats, treatment with antibiotics for 6 weeks increases DA content in PFCx, amygdala, and striatum (Hoban et al., 2016a). In this context, it has been shown that in the offspring of mice that received absorbable and non-absorbable antibiotics during gestation until lactation, there is an increase in serum corticosterone levels (Scheer et al., 2017). On the other hand, prenatal exposure to glucocorticoids has been shown to produce long-term increases in markers of the dopaminergic system in adult mice of both sexes, such as increased TH in substantia nigra and VTA, and increased D_2_ in NAcc and caudate putamen (CPu) (Virdee et al., 2014; Gillies et al., 2016). However, other markers exhibit sex differences due to prenatal glucocorticoid exposure, such as increased and decreased D1 expression in NAcc and CPu of females and males, respectively. Furthermore, prenatal glucocorticoid exposure decreased and increased DAT expression in NAcc and striatum of females and males, respectively (Virdee et al., 2014). Finally, in this work, an increase in dopamine release in NAcc of males exposed prenatally to glucocorticoids has been observed while it decreases in females (Virdee et al., 2014). These sexual differences have also been observed in our results: ELEA increased the expression of D_1_ and TH of NAcc in females (Figures 6B, 7B). In addition, in control animals, we observed sex differences in the expression of D_1_ (Figure 6B), D_2_ (Figures 6D–F), and TH (Figure 7B) in females compared to males. The evidence presented above strongly suggests an activation of the HPA axis as a sex-dependent mechanism mediated by ELEA, despite not having measured serum corticosterone levels. Further studies should be performed to test this theory.

Finally, we cannot rule out the possibility that the effects we observed are due to changes related to central delivery of the drug through the blood brain barrier caused by antibiotic exposure during early-life. On this remark, Leclercq et al. (2017) showed that treating pregnant BALB/c mice with penicillin in their drinking water, 1 week prior to parturition until weaning, results in 42-days old offspring with a differential increase in tight junction mRNA and protein expression in hippocampus but not in PFCx (Leclercq et al., 2017). Although this study differs from ours in rodent species, antibiotic type and treatment, and age of the studied offspring, it does suggest a regulation of antibiotic exposure in early life on blood-brain barrier permeability, which could affect psychostimulant drugs effect on the resulting animal. In addition, in our model of study, we cannot discard changes in gut microbial enrichment that could induced overpopulation of bacteria leading to increased permeability of the blood-brain barrier, affecting the absorption of abuse drugs such as DAT blockers into the CNS.

Overall, the differences that we observed may be related to sex hormones. Johnson et al. (2010a) showed that castrated male rodents (rats and mice) had lower counts of TH immunoreactive (TH-IR) cells in VTA and substantia nigra pars compacta, suggesting that testosterone may be suppressing and regulating midbrain dopaminergic pathways (Johnson et al., 2010a). On the other hand, ovariectomized female rodents also showed fewer TH-IR cells in both nuclei; however, when comparing the roles of estrogen receptors (ER) *α* and *β*, their results showed that female, but not male mice knockouts for ERα, displayed decreased levels of TH-IR in midbrain regions. Conversely, ERβ knockouts had TH-IR similar to wild-type animals (Johnson et al., 2010b), suggesting that female sex hormones also regulate the dopaminergic pathways in midbrain but in a more intricate manner, which may be in part responsible for differences observed in this study.

In conclusion, we have effectively demonstrated that early-life exposure to a non-absorbable broad-spectrum antibiotic cocktail alters the rat dopaminergic circuitry development resulting in a set of sex-dependent effects on drug response during adulthood. However, as DA neurotransmission not only depends on protein content, and it is not only present in the reward system, some questions also remain, such as how does *in-vivo* DA release occur under a DAT blocker challenge and evaluate locomotor activity on ELEA animals, respectively. Also, given that the antibiotic exposure was through oral administration to the pregnant rat, it is interesting to characterize the offspring’s gut microbiota composition in adulthood and evaluate possible interactions between microbial components and drug response. Lastly, as we observed the different effects of ELEA depending on the animal’s sex, it becomes imperative to assess sexual hormone contribution to ELEA’s response on the developing rat.

## Data Availability

All datasets generated for this study are included in the article and/or the Supplementary files.

## Abbreviations

CPP, Conditioned place preference; Ctrl, Control; CNS, Central nervous system; CPu, Caudate putamen; DA, Dopamine; DAT, Dopamine transporter; DOPAC, 3,4-dihydroxyphenylacetic acid; D1, Dopamine type 1 receptor; D2, Dopamine type 2 receptor; ELEA, Early-life exposure to antibiotics; ER, Estrogen receptor; ED, Embryonic day; FSCV, Fast-scan cyclic voltammetry; Fig, Figure; GN, Peptidoglycan; GF, Germ-free; i.p., Intraperitoneal; MPH, Methylphenidate; NAcc, Nucleus Accumbens; pDAT, Phosphorylated dopamine transporter; PFCx, Prefrontal cortex; PD, Parkinson’s disease; PD, Postnatal day; Supp, Supplementary; TH, Tyrosine hydroxylase; TH-IR, Tyrosine hydroxylase immunoreactive; VTA, Ventral tegmental area; 6-OHDA, 6-hydroxydopamine
