# Supplementary material for: Early-Life Exposure to Non-Absorbable Broad-Spectrum Antibiotics Affects the Dopamine Mesocorticolimbic Pathway of Adult Rats in a Sex-Dependent Manner
Source: Front Pharmacol. 2022 Jun 30;13:837652. doi: 10.3389/fphar.2022.837652 (PMC9280042; doi:10.3389/fphar.2022.837652)
Supplement: Supplementary file 2 [file DataSheet2.docx]

**Supplementary figures**


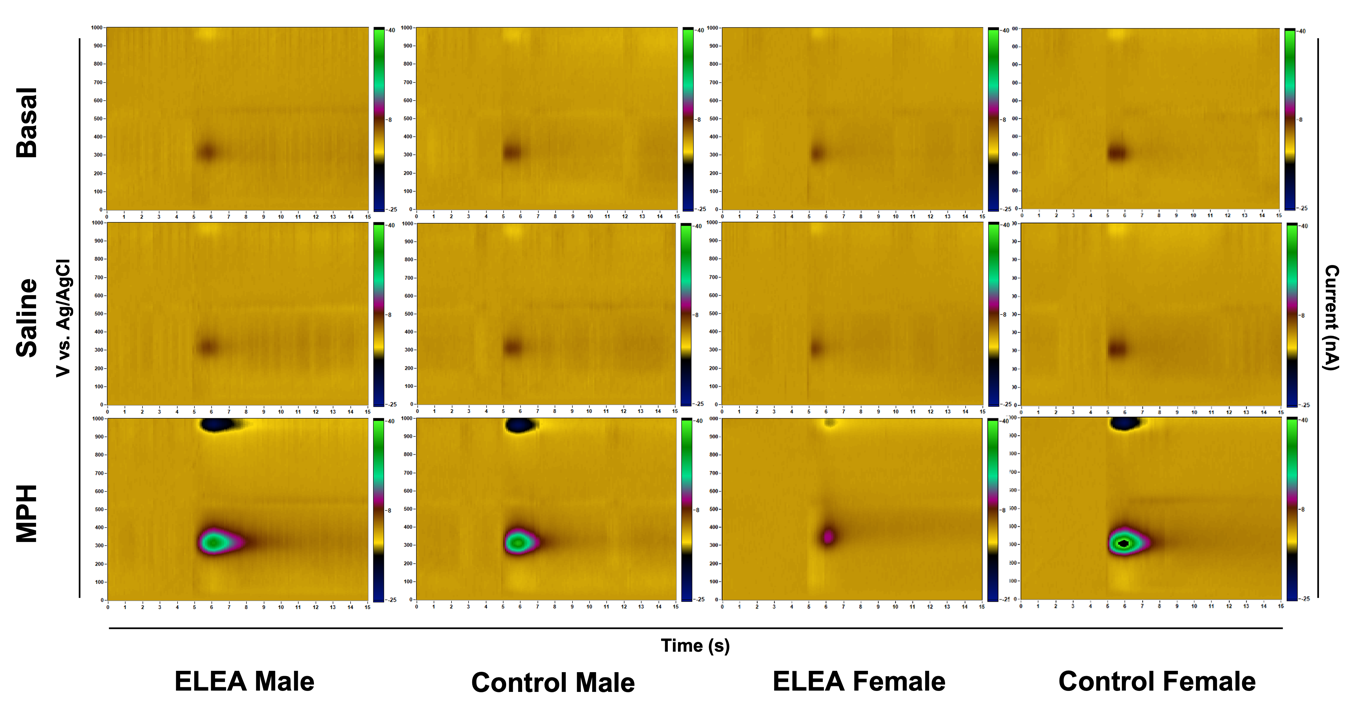
**Supp. Figure 1**

Color plots of representative peak high of striatal DA release evoked by electrical stimulation in basal, saline (1 mL/Kg, i.p.) and MPH (5 mg/Kg, i.p.) conditions. MPH color plot represents the highest peak registered, at 45 min.

**Supp. Figure 2
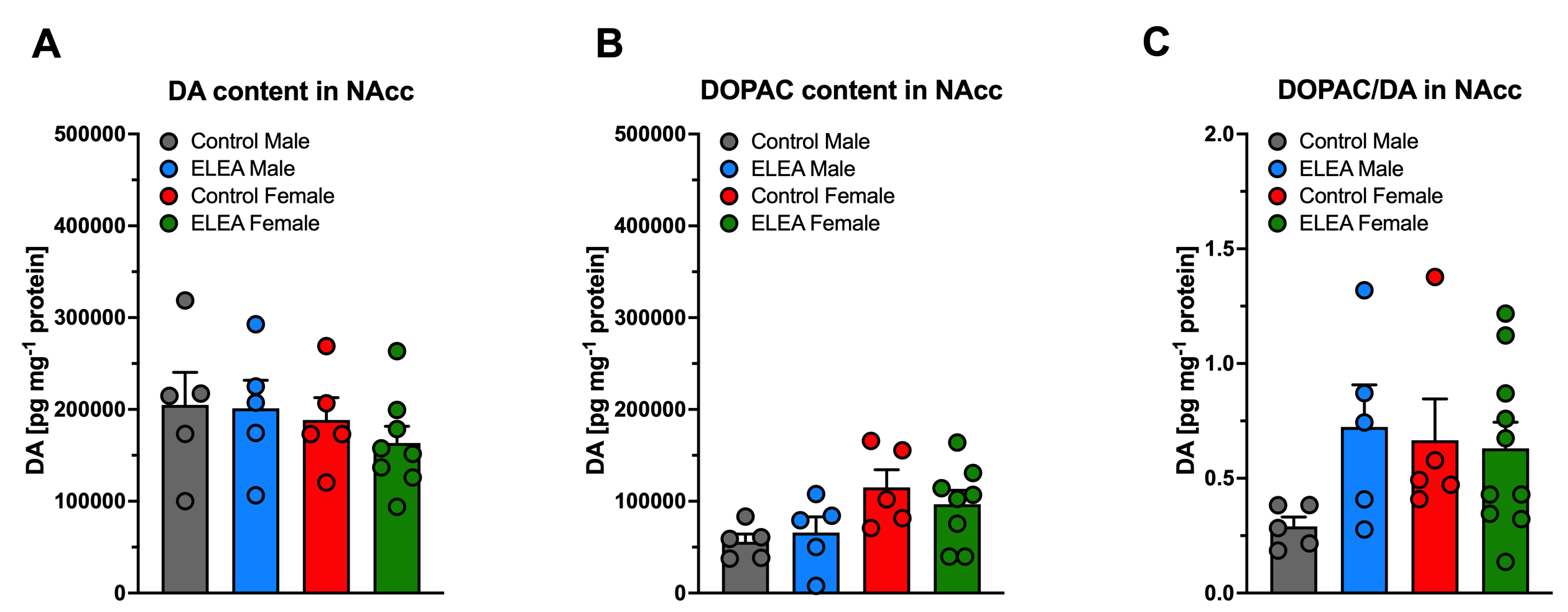
**

DA and DOPAC content of NAcc. There were no differences within the experimental groups.

**Supp. Figure 3**


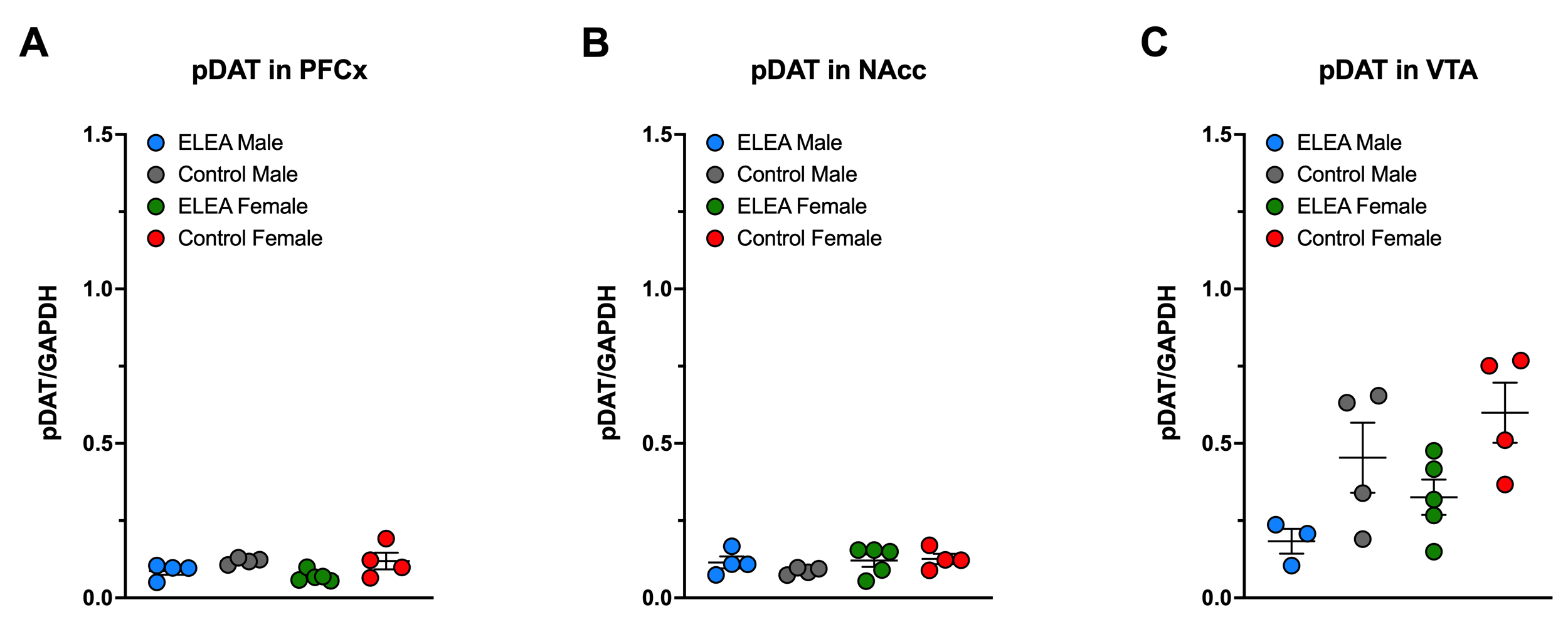

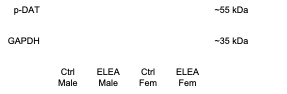

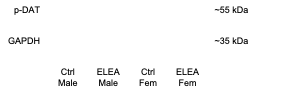

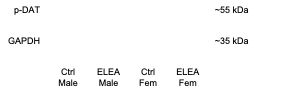

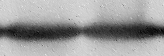

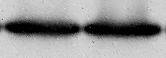

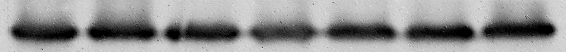

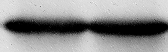

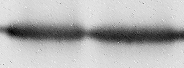

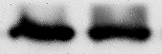

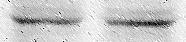

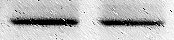

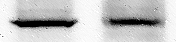

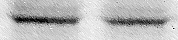

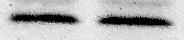

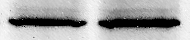

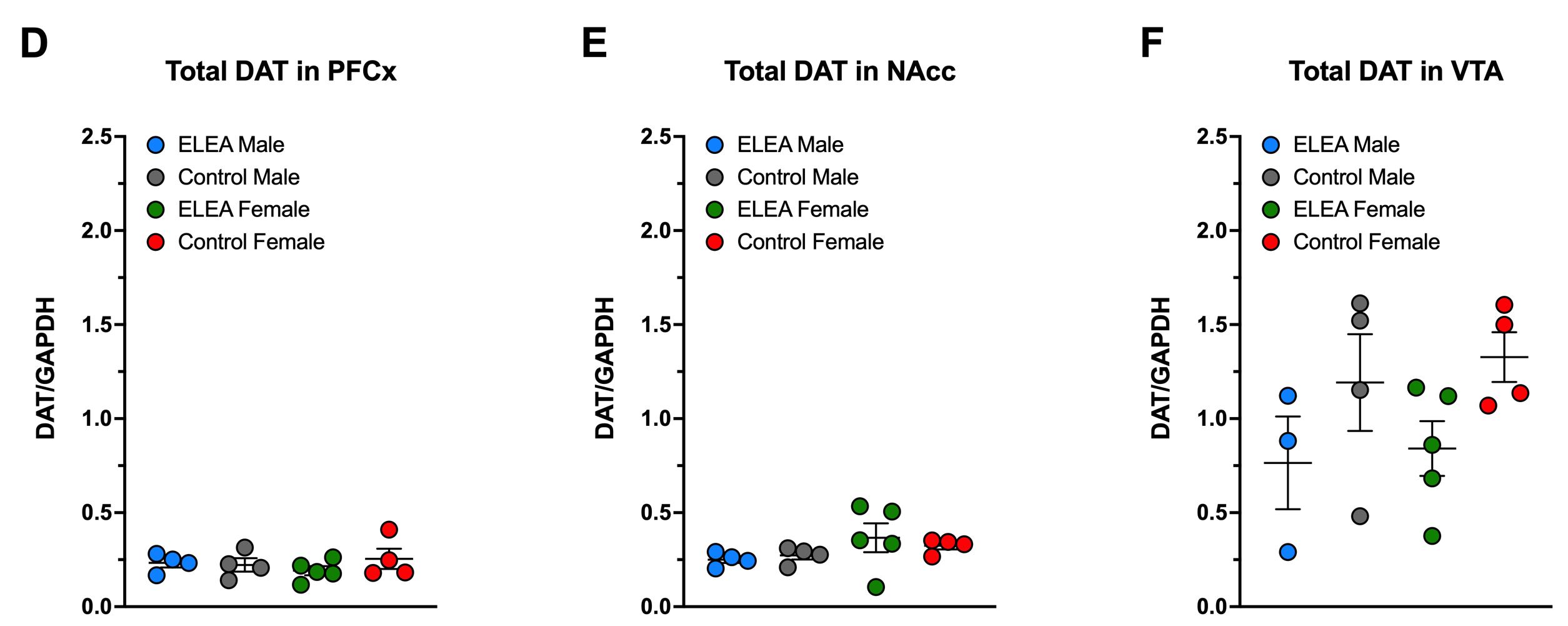

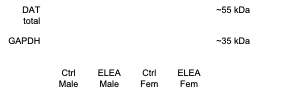

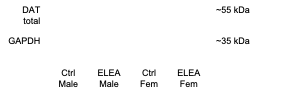

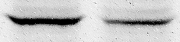

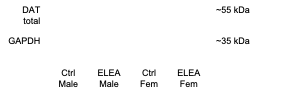

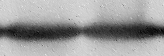

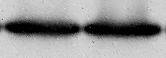

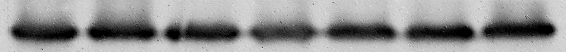

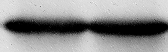

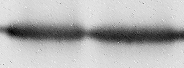

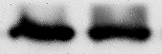

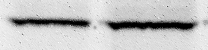

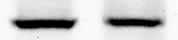

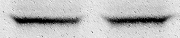

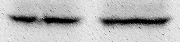

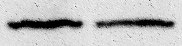


pDAT and total DAT protein levels expression from mesocorticolimbic system. No differences were observed in DAT expression within the mesocorticolimbic system.
